# CDKN2A Deletion in Melanoma Excludes T Cell Infiltration by Repressing Chemokine Expression in a Cell Cycle-Dependent Manner

**DOI:** 10.3389/fonc.2021.641077

**Published:** 2021-03-25

**Authors:** Zhen Zhu, Hao Song, Juan Xu

**Affiliations:** ^1^ The State Key Laboratory of Pharmaceutical Biotechnology and MOE Key Laboratory of Model Animals for Disease Study, Model Animal Research Center, Nanjing University, Nanjing, China; ^2^ Institute of Dermatology, Chinese Academy of Medical Sciences and Peking Union Medical College, Nanjing, China; ^3^ Department of Gynecology, Women’s Hospital of Nanjing Medical University (Nanjing Maternity and Child Health Care Hospital), Nanjing, China

**Keywords:** CDKN2A, T-cell infiltration, chemokine, melanoma, cell cycle dependent

## Abstract

T-cell-mediated immune response is the prerequisite for T-cell-based immunotherapy. However, the limitation of T-cell infiltration in solid tumors restricted the therapeutic effect of T-cell-based immunotherapy. The present study screened the molecular and genetic features of The Cancer Genome Atlas (TCGA)-skin cutaneous melanoma (SKCM) cohort, revealing that T-cell infiltration negatively correlated with genome copy number alteration. The analysis of the TCGA-SKCM cohort indicated that the copy number of CDKN2A was significantly decreased in patients with low T-cell infiltration. The results were validated in the other two melanoma cohorts (DFCI, Science 2015, and TGEN, Genome Res 2017). Besides, the immunohistochemistry analysis of CDKN2A and CD8 expression in 5 melanoma *in situ* and 15 invasive melanoma patients also showed that CD8 expression was decreased in the patients with low CDKN2A expression and there was a positive correlation between CDKN2A and CD8 expression in these patients. Interestingly, the CDKN2A deletion group and the group with low expression of T-cell markers shared similar gene and pathway alteration as compared with the normal CDKN2A group and the group with high expression of T-cell markers, especially the chemokine pathway. Further mechanistic study indicated that CDKN2A enhanced T cell recruitment and chemokine expression possibly through modulating MAPK and NF-κB signaling pathways in a cell cycle–dependent manner. Finally, we also found that CDKN2A deletion negatively correlated with the expression of T-cell markers in many other cancer types. In conclusion, CDKN2A deletion could inhibit T cell infiltration by inhibiting chemokine expression in a cell cycle dependent manner.

## Introduction

Cancer was described as a Darwinian evolutionary process driven by mutations (1–3). Mutagenesis in malignancies provides a pool for the immune system and selection of anticancer therapies (4). A tumor evolves from several single cells to malignant lesions through a series of mutations, thus acquiring the ability to fight against anticancer immune response and remodel the tumor microenvironment to immune tolerance (5–8). On the contrary, tumor cells possess a large number of neo-antigens as a result of genetic evolution, which may trigger the immune response in the tumor microenvironment (9–11). The immune system can inhibit tumor growth through cytotoxic-T-cell and cytokine-mediated lysis of tumor cells (12–14). However, the advantages in cell and tumor microenvironment enable tumor cells to evade immune surveillance by losing immunogenicity and acquire immunosuppressive characteristics (15–18). As a result, the immune system selectively repressed the pro-inflammatory genetic clones and retained the immunosuppressive ones, and thus forms the immune suppression signature. Exploring the origin of immunosuppression clones is a potential explanation for deportation T of cell infiltration in tumor microenvironment.

Cyclin-dependent kinase inhibitor CDKN2A is ubiquitously expressed in multiple cell types and served as tumor suppressor in tumor suppression. The CDKN2A locus encodes two gene products, p16INK4A and p14ARF, through alternative reading frames (ARFs) (19). Studies on human genetic variation demonstrated that p16INK4A deletion was the predominated event in cancer (20–22). p16INK4A exerts its tumor-suppressive function by inhibiting cyclin-dependent kinase CDK4 and CDK6 expression and preventing the phosphorylation of retinoblastoma (RB) protein at the G1/S cell cycle checkpoint (23). Tumors with p16INK4A deletion had superiority in overcoming cellular senescence and apoptosis compared with tumors with wild type p16INK4A, indicating that p16INK4A deletion gave cells an advantage in tumor evolution (24).

This study aims to find the genetic events and the underlying mechanism that may cause the immune suppressive signature. Our study found that CDKN2A deletion negatively correlated with the expression of T-cell markers and T-cell signature score in three human skin cutaneous melanoma cohorts. The ingenuity pathway analysis (IPA) of the differentially expressed genes and pathways between CDKN2A normal and deleted group as well as the patients with high or low expression of T cell markers showed that they share the similar pathway alteration, especially the chemokine pathway. Next, we found that CDKN2A could enhance chemokine expression in a cell cycle–dependent manner possibly by reducing p38/MAPK and NF-κB activation. Finally, we validated the relationship between CDKN2A deletion and CD8A as well as chemokine expression in urothelial bladder carcinoma (BLCA), pancreatic adenocarcinoma (PAAD), lung adenocarcinoma (LUAD), head and neck squamous cell carcinoma (HNSCC), stomach adenocarcinoma (STAD), lung squamous cell carcinoma (LSCC), and adrenocortical carcinoma (ACC) in the TCGA cohorts, and our results indicated that patients with CDKN2A deletion had lower T-cell infiltration and chemokine expression. Taken together, the study found an intrinsic genetic event, which is negatively associated with T-cell infiltration through reducing chemokine expression in tumor microenvironment.

## Materials and Methods

### Dataset Download and Preprocessing

The gene expression, genetic alternation, and clinical data of The Cancer Genome Atlas (TCGA)-skin cutaneous melanoma (SKCM) cohort were downloaded from Genomic Data Commons Data Portal (https://portal.gdc.cancer.gov/). The Fragments Per Kilobase per Million (FPKM) value of each transcript was first transformed into transcripts per million (TPM). The median expression was applied for one gene with multiple transcripts. The differentially expressed genes (DEGs) were generated with Limma (R-package) based on the log2 (TPM + 1) value. The gene expression, gene level copy number alternation, and clinical data of metastatic melanoma (DFCI, Science 2015) (25) and Acral Melanoma (TGEN, Genome Res 2017) (26) were collected from Cbioportal (https://www.cbioportal.org/) (27, 28). The median expression of datasets DFCI and TGEN was applied in the following analysis. The gene expression and copy number of bladder carcinoma (BLCA), pancreatic adenocarcinoma (PAAD), lung adenocarcinoma (LUAD), head and neck squamous cell carcinoma (HNSCC), stomach adenocarcinoma (STAD), lung squamous cell carcinoma (LSCC), and adrenocortical carcinoma (ACC) cohorts were downloaded from Cbioportal (https://www.cbioportal.org/) (27, 28). Log2 [RSEM (RNA-Seq by Expectation-Maximization) + 1] was applied for charting. The gene expression and copy number variation data of melanoma cell lines were collected from Cancer Cell Line Encyclopedia (CCLE, https://portals.broadinstitute.org/ccle).

### Dys-Regulated Genes

Differential expression of CD8 high (*n* = 117) and low (*n* = 117), CDKN2A diploid (*n* = 79), and CDKN2A deletion (*n* = 79) in the TCGA-SKCM cohort was detected from Limma (R-package) based on the log (TPM + 1) (log2 fold change >1; *P* value < 0.05; False Discovery Rate (FDR) < 0.01). A total of 79 patients with maximum copy number deletion were selected to generate DEGs of CDKN2A so as to equilibrate patient number with CDKN2A diploid. A total 566 and 1,199 genes were differeratially expressed in CDKN2A diploid/deletion and CD8A high/low, respectively.

### Ingenuity Pathway Analysis and Gene Set Enrichment Analysis

SKCM patients with CD8A high and low or CDKN2A normal and deletion were grouped and subjected to IPA and gene set enrichment analysis (GSEA). The data were analyzed using Qiagen’s IPA (Qiagen, Redwood City, http://www.qiagen.com/ingenuity). IPA software (Qiagen Inc., CA, USA) was used to discover relevant pathway alternations in different groups according to Fisher’s exact test enrichment statistics and Benjamini–Hochberg corrected *P*-value calculations.

The GSEA was performed using GSEA desktop application (https://www.gsea-msigdb.org/gsea/downloads.jsp). The C6 oncogenic signature gene sets were applied to estimate the pathway change in different groups.

### Cell Culture

Mouse melanoma cell line B16F0 cells were obtained from the Cell Bank of Type Culture Collection, Chinese Academy of Science (Shanghai, China) and maintained in Dulbecco’s modification of Eagle’s medium (DMEM) (SH30022, HyClone, China) supplemented with 10% Fatal Bovine Serum (FBS) (10099, Gibco, Australia) and penicillin/streptomycin (30-002-CI, Corning, CA, USA) at 37℃ in a humidified atmosphere with 5% CO_2_.

### Vector Construction and Lentivirus Transduction

The sequence with proximal promoter (−445 to +271) and the coding sequence of CDKN2A (p16INK4 open reading frame) was cloned into pCDH-CMV vector. And then the 293T cells were co-transfected with the lentivirus packaging vectors as well as the p16INK4 expression vector. Virus supernatants were harvested 72 h after transfection, filtered, and concentrated. The B16F0 cells were transfected with the lentivirus with proximal promoter drived CDKN2A. And the positive clones were selected after 1 µg/ml of puromycin treatment (P8833, Sigma–Aldrich, USA) for 3 days.

### RNA Isolation and Quantitative Real-Time PCR

Total RNAs were isolated from cells using TRIzol reagent (15596018, Invitrogen). Then, 1 μg RNA was reverse-transcribed to cDNA following the manufacturer’s protocol (RR047A, TaKaRa, China). The gene expression level was determined by qPCR using an SYBR Premix Ex Taq I and II kit (DRR420A, TaKaRa) in an ABI 7500 StepOne Plus Real-Time PCR instrument (Applied Biosystem, NY, USA). The expression of all genes was normalized to actin, and the relative expression of each gene was determined in ABI StepOne Plus software by the 2^–ΔΔct^ method. Each experiment was repeated at least three times. All the primers for RT-qPCR are listed in **Supplementary Table S1**.

### Western Blot

Total protein was extracted using RIPA lysis buffer supplemented with a phosphatase inhibitor PhosStop mini-tablet (4906845001, Roche Diagnostics, IN, USA) and a protease inhibitor cocktail mini-tablet (4693159001, Roche Diagnostics). After isolation, total protein (20 μg) was separated by 8%–12% dodecyl sulfate sodium salt -Polyacrylamide gel electrophoresis (SDS-PAGE) and transferred onto a 0.45-μm polyvinylidene-difluoride (PVDF) membrane (05317-10EA, Millipore, MA, USA). The membrane was blocked with 5% nonfat milk in TBST and incubated with a primary antibody against Vinculin (1:1000, sc-73264, RRID : AB_1131292, Santa Cruz, USA), phospho-p65 (Ser536) (1:1000, #3031S, RRID : AB_330559, Cell Signaling Technology, Boston, USA), phospho-p38 MAPK (Thr180/Tyrl82) (1:1000, #4631S, RRID : AB_331765, Cell Signaling Technology), phospho-ATF2 (T71) (1:1000, BS4018, RRID : AB_1664091, Bioworld, China), phospho-p44/42 MAPK (ERK1/2) (Thr202/Tyr204) (1:1000, #4370P, RRID : AB_2315112, Cell Signaling Technology), and IκBα (1:1000, #9242S, RRID : AB_331623, Cell Signaling Technology), CDK4 (1:500, sc-23896, RRID : AB_627239, Santa Cruz), E2F1 (1:1000, 12171-1-AP, RRID : AB_2096958, Proteintech), p16INK4 (1:500, sc-1661, RRID : AB_628067, Santa Cruz), Phospho-Histone H3 (1:1000, #9701, RRID : AB_331535, Cell Signaling Technology), Cyclin D1 (1:200, sc-753, RRID : AB_2070433, Santa Cruz), Cyclin E1 (1:500, BS1086, RRID : AB_1663604, Bioworld), Cyclin B1 (1:1000, sc-245, RRID ;: AB_627338, Santa Cruz), at 4°C overnight. On the other day, the membranes were washed with Tris Buffered Saline Tween ^®^ 20 (TBST) thrice and incubated with Horseradish Peroxidase (HRP)-conjugated anti-rabbit or anti-mouse antibodies (1:10000, G-21040/G-21234, RRID : AB_2536527/RRID : AB_2536530, Thermo Fisher Scientific, USA) for 1 h at room temperature. Western blots were visualized using enhanced chemiluminescence reagents (WBKLS0500, Millipore, USA).

### Enzyme-Linked Immunosorbent Assay

About 1 × 10^5^ cells were seed in 6-well dish with 2 ml complete medium. After 24 h culture with or without treatment, conditional medium was collected and the protein expression of Ccl4 (70-EK262, MultiSciences, China), Ccl5 (FMS-ELM106, FcMACS, China), and Cxcl10 (EK2682/2, MultiSciences) expression in medium were detected by the ELISA kit provided above according to manufacturer’s protocol.

### Clinical Sample Collection

Clinical samples in this study were collected from patients diagnosed with *in situ* (n = 5) or invasive (n = 15) melanoma undergoing surgical resection in the Pathology Department, Institute of Dermatology, Chinese Academy of Medical Sciences and Peking Union Medical College from 2018 to 2020. The samples were staged according to the American Joint Committee on Cancer (AJCC) melanoma staging system (The Seventh Edition). All samples were reviewed and approved by the Ethical Committee of Institute of Dermatology, Chinese Academy of Medical Sciences and Peking Union Medical College. Samples were fixed in 10% formalin, dehydrated by ascending grades of ethanol, immersed in xylene and then embedded in paraffin wax.

### Immunohistochemistry

Immunohistochemical staining was performed on formalin-fixed, paraffin-embedded 4-µm thick tissue sections, which were dewaxed with xylene and rehydrated by passage through a graded alcohol series. As most of melanoma tissues were rich in melanin, depigmentation of melanin was done by 10% H_2_O_2_ solution for 1 h at 56°C. Antigen epitope retrieval was performed using EDTA buffer (pH 8.0) (DAKO, Hamburg, Germany) for 15 min at 96°C. This procedure was followed by 1-h incubation at room temperature with CDKN2A antibody (1:100, sc-56330, RRID : AB_785018, Santa Cruz) or CD8 antibody (DAKO, ready-to-use, Hamburg, Germany) in a humidified chambers. Tissues incubated with PBS instead of the primary antibody served as the negative control, whereas tissues incubated with Melan-A (DAKO, ready-to-use, Hamburg, Germany) served as the positive control. The sections were washed three times (5 min each) with PBS and then incubated with HRP-labeled secondary antibody for 15 min at room temperature. After three washes (5 min each) with PBS, the sections were stained using a peroxidase detection system. Antibody binding was visualized using diaminobenzidine (Sigma-Aldrich) as chromogen, and counterstaining was done with hematoxylin.

The results for CDKN2A and CD8 expression were quantified blindly and independently by two independent observers. Disagreements among observers were discussed under a multi-scope microscope for a final consensus. The mean percentage of CKDN2A positive cells was determined in at least four random areas at a magnification of 400×. Each value was assigned to one of five categories (0, none; 1, <25%; 2, 26–50%; 3, 51–75%; 4, >75%). The intensity of CKDN2A immunostaining was scored as negative (0), weak (1), moderate (2), and intense (3). The scores for staining intensity and percentage of positive cells were multiplied to produce a final score for each case. The final score greater than 2 was defined as high expression. The value of CD8 positive cells was assigned to three levels, as none (0), locally in the tumor tissue (1), abundant in the tumor tissue (2).

### Cell Synchronization and Cell Cycle Analysis

Cell synchronization was blocked with double thymidine (T9250, Sigma, Shanghai, China). The cells were plated and treated with thymidine (2mM) for 18 h twice. And then the cell cycle was analyzed after thymidine removing for 0, 2, 6, 8, 10, 12, 14, and 24 h. The cells were fixed with 70% ethanol, RNA was removed with RNase A, and the cells were stained using Propidium Iodide (PI) (50 μg/ml). The fluorescence of PI was detected using a BD FACS Calibur system (BD Biosciences, CA, USA). Thymidine was released for 4 h to obtain G2/M-phase cells.

### Statistical Analysis

The unpaired-sample *t* test was used to determine significant differences among variables and groups. The correlation of gene expression with genetic events was determined using Pearson correlation, Fisher’s exact test, or *χ*² test. Kaplan–Meier survival curves with the log-rank test were used to analyze the overall survival of each dataset. The correlation of protein expression and clinicopathological characteristics was analyzed by Mann-Whitney U test by SPSS 22.0. R-package “survival” was determined using Cox proportional hazards regression in univariate and multivariate models. Statistical analyses were performed in GraphPad Prism 8.0 (GraphPad Software Inc, USA). Semi-quantification of protein expression was determined by digitally captured images using the NIH Image J software. Data were presented as mean ± standard deviation with at least three biological independent experiments (^*^ *P* < 0.05; ^**^ *P* < 0.01; ^***^ *P* < 0.001. In all analyses, *P* < 0.05 indicated a statistically significant difference.

## Results

### Analysis of Genetic Events Associated With CD8+ T-Cell Infiltration

**Figure 1:**
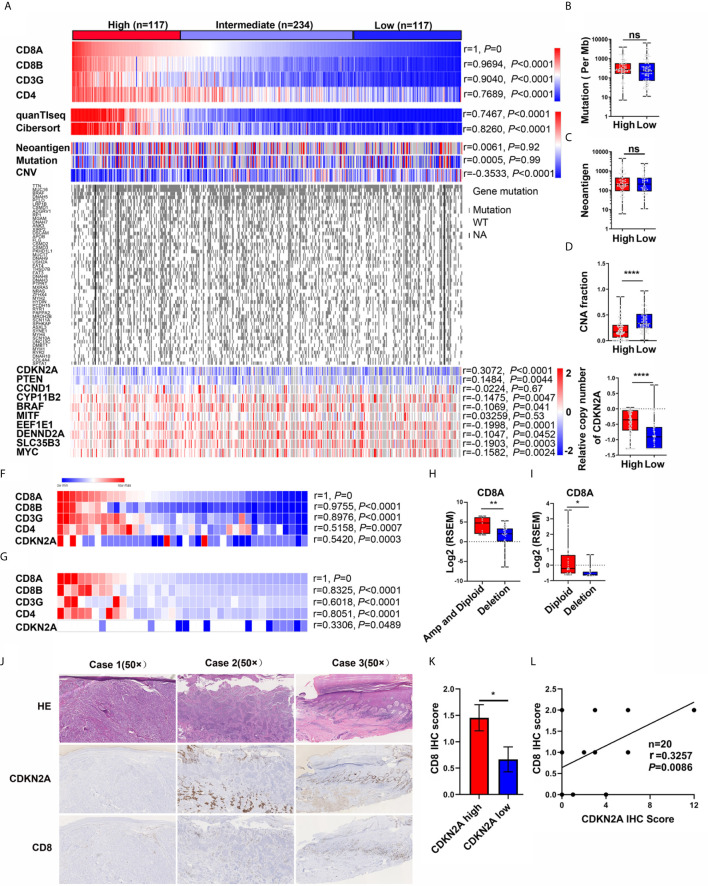
Discovery of the genetic events correlated with CD8+ T cell infiltration in melanoma. **(A)** Heatmap of T cell marker expression, CD8+ T cell score, the genetic events, the top 50 genes with the highest mutation rate, and the top 10 genes with the highest copy number alteration in TCGA-melanoma cohort. Pearson correlation analysis between CD8A expression and other T cell marker expression, CD8+ T cell score, genetic events, top 10 genes with the highest copy number alteration were also analyzed. **(B–E)** The difference of mutation **(B)**, neoantigen **(C)**, CNA fraction **(D)**, and CDKN2A copy number **(E)** in CD8A High and Low subgroups. **(F)** Heatmap of CD8+ T cell marker expression and CDKN2A copy number in DFCI cohort (n = 40). Pearson correlation analysis indicated that the copy number of CDKN2A is positively associated with CD8+ T cell marker expression. **(G)** Heatmap of CD8+ T cell marker expression and CDKN2A copy number in TGEN cohort (n = 36). Pearson correlation analysis indicated that the copy number of CDKN2A is positively associated with CD8+ T cell marker expression. **(H)** CD8A expression in melanoma patients with diploid and amplificated CDKN2A (n = 9) compared with patients with CDKN2A deletion (n = 31) (DFCI cohort). RSEM, RNA-Seq by Expectation-Maximization. **(I)** CD8A expression in melanoma patients with diploid or amplificated CDKN2A (n = 24) compared with patients with CDKN2A deletion (n = 12) (TGEN cohort). RSEM, RNA-Seq by Expectation-Maximization. **(J)** The representative IHC analysis of CDKN2A and CD8 expression in melanoma patients. (Case 1: Invasive melanoma with negative CDKN2A expression and few CD8-positive lymphocytes. Case 2: Invasive melanoma with positive CDKN2A expression in the deep dermis, but loss of CDKN2A expression in the upper dermal component, while few CD8-positive lymphocytes in the upper dermal tumor tissue and relatively high CD8-positive lymphocytes in the deep dermis. Case 3: melanoma *in situ* with positive CDKN2A expression, while abundant CD8-positive lymphocytes in the papillary layer of dermis.) **(K)** The difference between the IHC score of CD8 in the melanoma patients with low CDKN2A expression (IHC score: 0~2) or high CDKN2A expression (IHC score: 3~12). **(L)** The pearson correlation coefficient analysis of CDKN2A and CD8 expression in 20 melanoma patients. *p < 0.05, **p < 0.01, ****P < 0.0001, ns, not significant.

The mRNA expression of T-cell markers (CD8A, CD8B, CD3G, and CD4) (29–31) in 468 melanoma samples from the TCGA-SKCM dataset was first analyzed to screen genetic events correlated with T-cell infiltration (**Figure 1A**), the study found that the expression of T-cell makers significantly correlated with the CD8+ T-cell signature score [CIBERSORT (32, 33) and quanTIseq (34)]. Then, the association between T-cell marker expression and genetic events, including neoantigen, mutation, and copy number alternation, was analyzed (**Figure 1A**). The results indicated that the expression of T-cell markers in melanoma showed no significant correlation with the mutation rate and neoantigen number (**Figure 1A**), but significantly correlated with copy number alteration (**Figure 1A**, Pearson’s *r* = −0.3533, *P* < 0.0001). Besides, gene copy number values negatively correlated with CD8A expression, indicating that a high copy number alternation was highly associated with CD8A expression (Pearson’s *r* = −0.9, *P* < 0.0001, gene number = 24,687) (**Figure 1A** and **Supplementary Figure S1A**). Next, patients in the SKCM cohort were divided into high (*n* = 117), intermediate (*n* = 234), and low (n = 117) groups based on the expression of T-cell makers and CD8+ T-cell signature score (**Supplementary Figure S1B**). No significant difference was found in the neoantigen number and mutation rate between T-cell high and low groups (**Figures 1B, C**), while the copy number alteration was significantly higher in the T-cell high group (**Figure 1D**). Further, the correlation between T-cell marker expression and the top 50 genes with the highest mutation rate and the top 10 genes with the highest copy number alteration was analyzed. No gene mutation was associated with T-cell marker expression. In contrast, the copy number variation of CDKN2A, PTEN, CYP11B2, BRAF, EEF1E1, DENND2A, SLC35B3, and MYC was significantly associated with T-cell marker expression; the correlation was the highest between CDKN2A copy number variation and T-cell marker expression in the TCGA-SKCM cohort (**Figures 1A, E**). And the T-cell signature score was significantly lower in patients with deleted CDKN2A than in patients with normal CDKN2A melanoma (*P* < 0.0001, **Supplementary Figure S1C**).

Independent cohorts DFCI (*n* = 40) and TGEN (*n* = 36) were used to confirm the results that CDKN2A loss was negatively associated with T-cell infiltration. The correlation analysis showed that the T-cell marker expression was positively associated with the CDKN2A copy number value (Pearson’s r = 0.5420 and *r* = 0.3306, **Figures 1F, G**). Furthermore, CD8A expression was significantly higher in CDKN2A normal patients than that in CDKN2A-deleted patients in these two cohorts (**Figures 1H, I**).

In the analyses of all three datasets, the copy number of CDKN2A positively associated with CD8A expression and CD8+ T-cell signature score. Specifically, the shallow and deep deletions of CDKN2A were identified as a feature of CD8A loss in melanoma.

To further explore the relationship between CDKN2A and CD8 in melanoma, we also analyzed p16^INK4^ (the protein product of CDKN2A) and CD8 expression by immunohistochemistry (IHC) in 5 melanoma *in situ* and 15 invasive melanoma samples. As shown in **Figure 1J** and **Supplementary Table S2**, CDKN2A was lost in 1/5 of melanoma *in situ* and 6/15 of invasive melanoma samples, which is consistent the previous report that loss of CDKN2A is associated with the invasive behavior of melanoma (35). Interestingly, the IHC score of CD8 was significantly decreased in melanoma patients with low expression of CDKN2A (IHC score: 0~2) as compared with the melanoma patients with high expression of CDKN2A (IHC score: 3~12) (**Figure 1K**). And the Pearson correlation analysis also showed that CDKN2A expression was positively associated with CD8 expression (**Figure 1L**, Pearson’s *r* = 0.3257, *P* = 0.0086).

### CDKN2A Diploid Patients Shared Similar Signatures With CD8A High Patients

**Figure 2:**
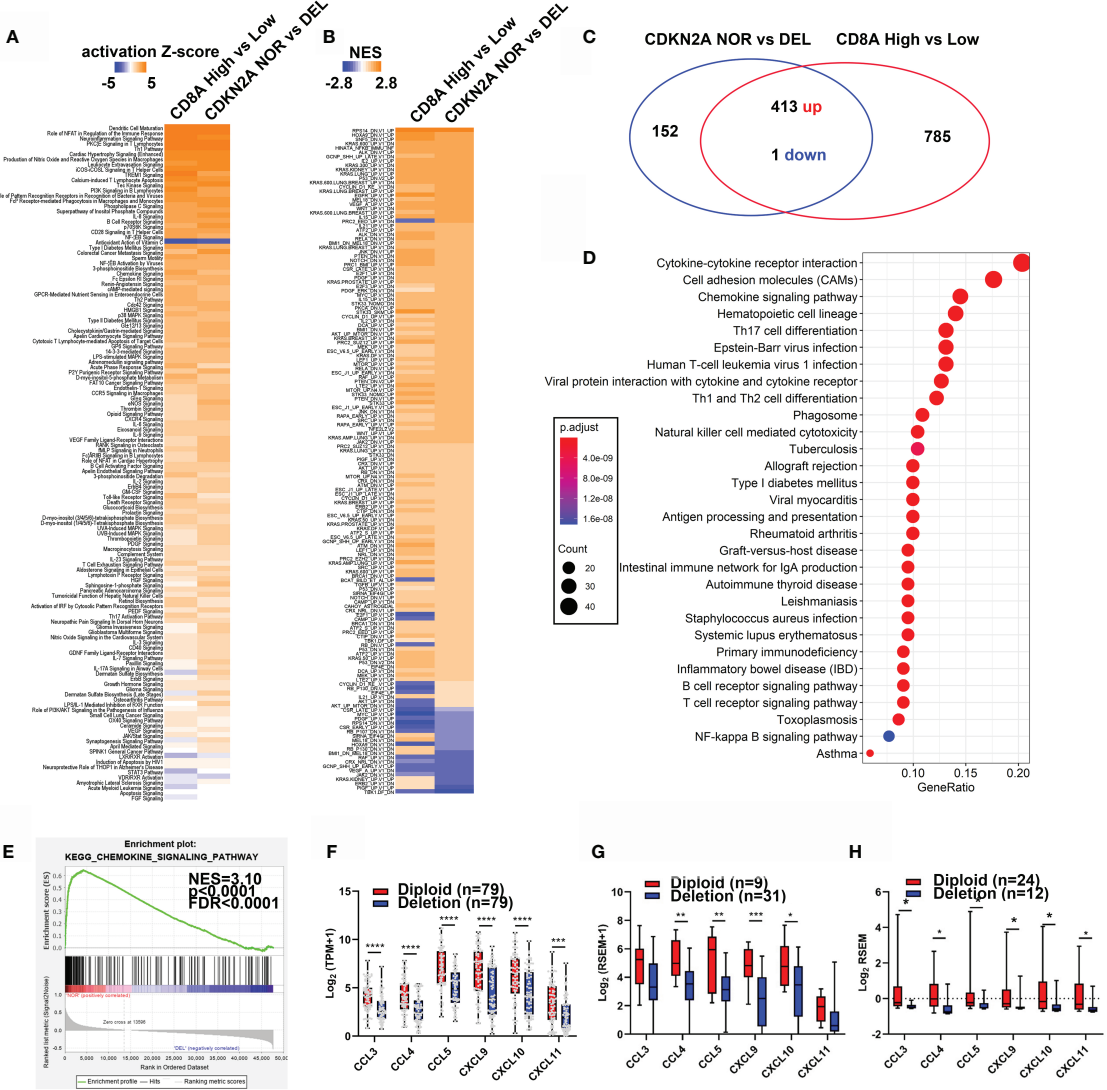
The patients with normal CDKN2A or high CD8A expression showed similar regulation of chemokine signaling pathway. **(A, B)** Heatmap analysis of the pathway enrichment partem of the differentially expressed genes in CD8A high/low groups (From **Figure 1**) and CDKN2A NOR (diploid, n = 79) and DEL (Deep deletion, n = 79) in IPA analysis **(A)** and C6 oncogenic pathway GSEA analysis **(B)**. **(C)** Venn diagram shows differentially expressed gene (DEG) number in CDKN2A NOR/DEL group and CD8A High/Low group. **(D)** KEGG analysis of common regulated gene in CDKN2A NOR/DEL and CD8A HIGH/LOW group. **(E)** GSEA analysis of the chemokine signaling pathway expression in CDKN2A NOR/DEL group. **(F)** The expression of known T-cell recruitment chemokines expression in CDKN2A normal and deleted group in TCGA-melanoma cohort. **(G)** The expression of known T-cell recruitment chemokines expression in CDKN2A normal and deleted group in DFCI-melanoma cohort. **(H)** The expression of known recruitment chemokines expression in CDKN2A normal and deleted group in TGEN-melanoma cohort. *p < 0.05, **p < 0.01, ***p < 0.001, ****P < 0.0001.

IPA and GSEA analysis of the dys-regulated genes (DEGs) between patients with CD8A high (*n* = 117) or low (*n* = 117) (**Figure 1A**), as well as in the patients with normal (NOR, *n* = 79) or deleted (DEL, *n* = 79) CDKN2A, in the TCGA-melanoma cohort were performed to determine whether CDKN2A was the driver gene for the regulation of T-cell infiltration. Interestingly, DEGs between the CD8A high and low groups shared the canonical IPA and GSEA results with DEGs between the CDKN2A normal and deleted groups (**Figures 2A, B**), indicating that CDKN2A might participate in T-cell infiltration in the TCGA-melanoma cohort. Besides, the Kyoto Encyclopedia of Genes and Genomes (KEGG) pathway analysis of the 413 genes that were consistently upregulated in the CDKN2A diploid group and the CD8A high group showed significant enrichment of immune cell response and immune cell migration pathway (**Figures 2C, D**). Among these, the cytokine–cytokine receptor interaction and chemokine signaling pathway were the most enriched pathways (FDR <4.0e-9, **Figure 2D**). Cytokines and chemokines are important regulators of T-cell infiltration. The study next assessed the expression of known T-cell recruitment chemokines in TCGA-melanoma and TGEN-melanoma cohorts. It showed that chemokines C-C Motif Chemokine Ligand 3 (CCL3), CCL4, CCL5, C-X-C Motif Chemokine Ligand 9 (CXCL9), CXCL10, and CXCL11 were significantly upregulated in the normal CDKN2A group than in the CDKN2A-deleted group (**Figures 2E–H** and **Supplementary Figures S2A** and **S2B**), indicating that CDKN2A might participate in the regulation of cytokine–cytokine receptor interaction or chemokine signaling pathway and thus contribute to T-cell recruitment in melanoma.

### CDKN2A Enhanced Chemokine Expression

**Figure 3:**
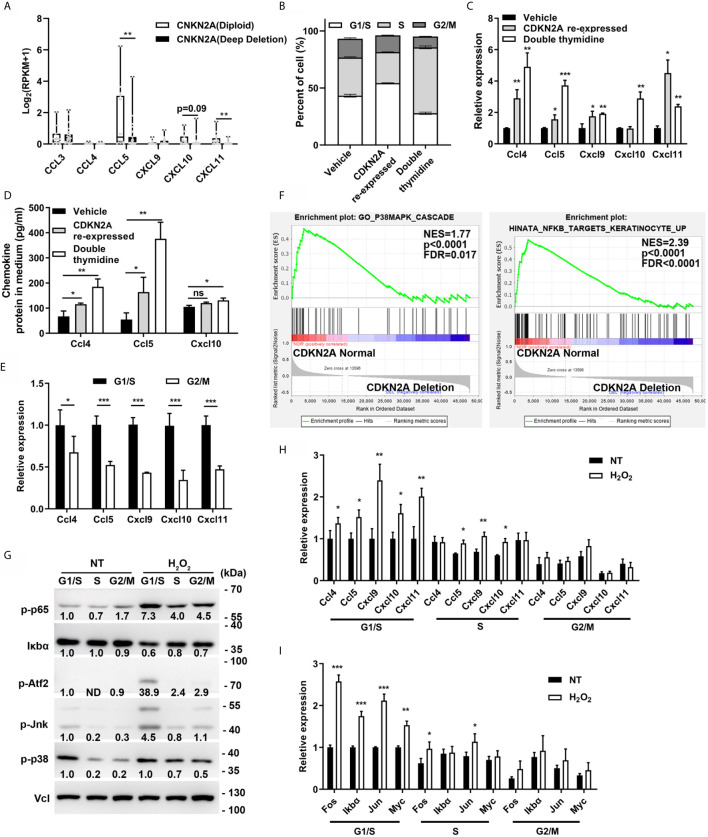
CDKN2A enhances chemokine expression. **(A)** Chemokine expression in CDKN2A Diploid and Deep deletion melanoma cell lines. **(B)** Cell cycle analysis of vehicle, double thymidine treated B16F0 cells and CDKN2A re-expressed B16F0 cells. **(C)** The mRNA expression of chemokine Ccl4, Ccl5, Cxcl9, Cxcl10, and Cxcl11 expression in control, CDKN2A re-expressed and double thymidine treated B16F0 cells. **(D)** ELISA analysis of the chemokine Ccl4, Ccl5, and Cxcl10 expression in control, CDKN2A re-expressed and double thymidine treated B16F0 cells. **(E)** The mRNA expression of chemokine Ccl4, Ccl5, Cxcl9, Cxcl10, and Cxcl10 in B16F0 cells arrested at G1/S and the cells arrested at G2/M phase. **(F)** The GSEA analysis of the gene expression in P38/MAPK and NF-κB pathway in patients with normal CDKN2A or CDKN2A deletion in TCGA-SKCM cohort. **(G)** The expression of protein in p38/MAPK and NF-κB pathway in B16F0 cells arrested at G1/S phase, S phase, G2/M phase with or without H_2_O_2_ treatment. **(H)** mRNA expression of chemokine Ccl4, Ccl5, Cxcl9, Cxcl10, and Cxcl11 in B16F0 cells treated with H_2_O_2_ or not. **(I)** The mRNA expression of genes downstream of p38/MAPK and NF-κB pathway in B16F0 cells arrested at G1/S phase, S phase, G2/M phase with or without H_2_O_2_ treatment. *p < 0.05, **p < 0.01, ***P < 0.001, ns, not significant.

Chemokine expression was first analyzed in the melanoma cell lines [from Broad Institute Cancer Cell Line Encyclopedia (CCLE)] with normal CDKN2A (diploid) or deleted CDKN2A to further explore the relationship between CDKN2A and chemokine expression. The results indicated that the chemokine CCL5 and CXCL11 expression was significantly decreased in CDKN2A-deleted melanoma cell lines (**Figure 3A**). B16F0 cells were described as CDKN2A null cell line (36). The CDKN2A expression was restored in B16F0 cells by transfection with the lentivirus containing proximal promoter-drived p16INK4 (the protein product of CDKN2A) expression. Previous study indicated that CDKN2A mainly regulate cell cycle progression by interacting with CDK4 and inhibit the function of retinoblastoma protein, thereby arresting the cell cycle at the G1/S phase (23, 37, 38). In this study, we found that the cell cycle protein Cyclin D1, Cyclin E1, CDK4, which are important for G1/S transition, were significantly increased, and the cell cycle protein Cyclin B1, Phospho-Histone H3, E2f1, which are important for G2/M transition, were significantly decreased in CDKN2A re-expressed B16F0 cells, indicating a G1/S phase arrest in these cells (**Supplementary Figure S3A**). So we hypothesized that CDKN2A enhance chemokine expression in a cell cycle–dependent manner. To confirm this, double thymidine treatment was employed to mimic the function of CDKN2A and arrest cell cycle in G1/S phase (**Figure 3B** and **Supplementary Figure S3B**). The results showed that the mRNA expression of Ccl4, Ccl5, Cxcl9, Cxcl10, and Cxcl11 and the protein expression of Ccl4, Ccl5, and Cxcl10 were significantly increased in Cdkn2a re-expressed and double thymidine treated B16F0 cells (**Figures 3C, D**). Then the chemokine expression was analyzed in the cells arrested in G1/S phase or released to G2/M phase to exclude the effect of double thymidine on chemokine expression. The results showed that chemokines were highly expressed only in G1/S-arrested melanoma cells, but not in the cells released to G2/M phase (**Figure 3E**), indicating that arresting cell cycle at G1/S phase by Cdkn2a or double thymidine can enhance those chemokine expression.

Chemokine expression is mainly regulated by NF-κB and p38/MAPK pathways (39, 40). Cellular stimulation, such as pro-inflammation cytokine (TNF-α), immunosuppression cytokine (TGF-β), and reactive oxygen species (ROS), has been found to induce chemokine expression through activating NF-κB and p38/MAPK pathways (41–44). Interestingly, we noticed that p38 and p65 were also obviously activated in H_2_O_2_, TNF-α, and TGF-β treated B16F0 cells as compared with control, and Ccl4, Ccl5, Cxcl9, and Cxcl10 expression were also significantly increased in H_2_O_2_ treated cells (**Supplementary Figures S3C** and **S3D**). Besides, supplementation of p38/MAPK inhibitor (SB203580) and NF-κB inhibitor (Caffeic Acid Phenethyl Ester, CAPE) significantly inhibited the H_2_O_2_ induced expression of Ccl4, Ccl5, Cxcl9, and Cxcl10 (**Supplementary Figure S3D**) and ectopic expression of transcription factor Atf2, which is an important target of p38/MAPK, also increased Ccl4, Ccl5, Cxcl9, and Cxcl10 expression (**Supplementary Figure S3E**). These results is consistent with previous results which indicated longer and stronger NF-κB response in the cells arrested in G1/S phase and delayed NF-κB response in the cells arrested in S phase, through NF-κB and E2F interaction (45, 46). As expected, CDKN2A loss resulted in decreased p38/MAPK and NF-κB signature score in TCGA-SKCM cohort as compared with CDKN2A normal group (**Figures 2A, B** and **3F**), indicating a negative association between CDKN2A deletion and p38/MAPK and NF-κB activation. Hence, we hypothesized that the association between CDKN2A and chemokine signature score was related to p38/MAPK and NF-κB pathway. Further study indicated that p38/MAPK and NF-κB signaling pathway was also higher in the B16F0 cells arrested at G1/S phase as compared with the cells released to G2/M phase, besides, H_2_O_2_ treatment also significantly increased the protein expression in 38/MAPK and NF-κB signaling pathway as well as the mRNA expression of the chemokine Ccl4, Ccl5, Cxcl9, and Cxcl10 in the B16F0 cells arrested at G1/S phase, however, the activation effect was decreased in the cells at G2/M phase, indicating that p38/MAPK and NF-κB pathway as well as the chemokine expression was enhanced in the cells arrested at G1/S phase (**Figures 3G, H**). The expression of p38/MAPK and NF-κB target genes, such as Fos, Jun, IκBα, and Myc, also significantly increased in the G1/S phase, mildly increased in the S phase, and did not change in the cells released to G2/M phase after H_2_O_2_ treatment in the B16F0 cells (**Figure 3I**). Taken together, the study found that arresting cell cycle at G1/S by CDKN2A increased chemokine expression through enhancing p38/MAPK and NF-κB pathway activation.

### CDKN2A Loss Correlated With CD8+ T-Cell Score in Other Solid Tumors

**Figure 4:**
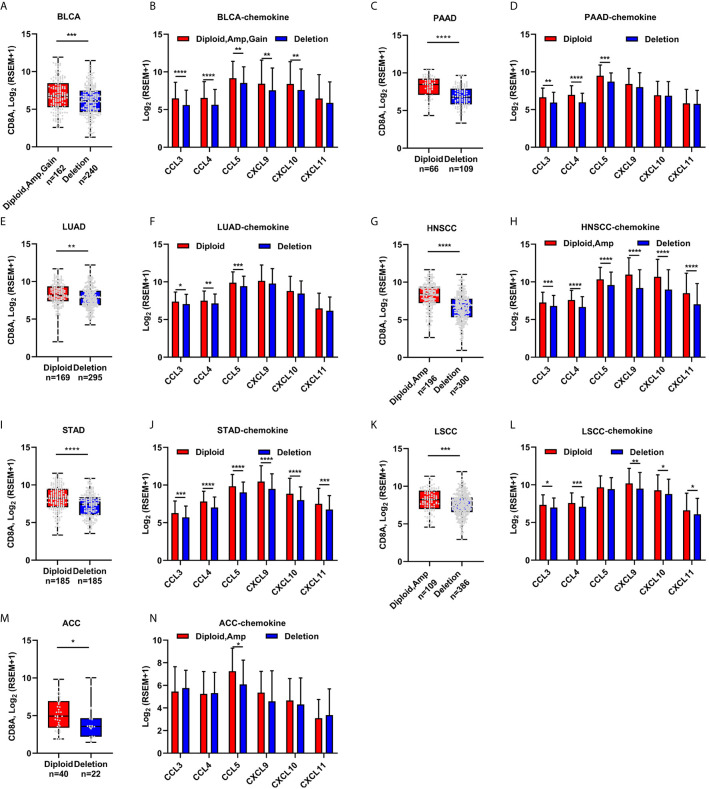
The correlation of CDKN2A deletion and CD8A expression, T cell attracted chemokine expression in other cancers. **(A–N)** CD8A and the known chemokine expression in Urothelial Bladder Carcinoma (BLCA) patients with normal and/or amplificated/gained and deleted CDKN2A **(A, B)**, in Pancreatic adenocarcinoma (PAAD, **C, D**), in Lung Adenocarcinoma (TCGA-LUAD, **E, F**), in Head and neck squamous cell carcinoma (HNSCC, **G, H**), Stomach Adenocarcinoma (STAD, **I, J**), in Lung squamous cell carcinoma (LUSC, **K, L**), and in Adrenocortical carcinoma (ACC, **M, N**). *p < 0.05, **p < 0.01, ***p < 0.001, ****P < 0.0001.

CDKN2A enhanced chemokine expression through a canonical pathway. Hence, it was proposed that CDKN2A regulated CD8+ T-cell infiltration in a cancer type–independent manner. Several cancer types, including urothelial BLCA, PAAD, LUAD, HNSCC, STAD, LSCC, and ACC, from the TCGA cohort were analyzed. Interestingly, CDKN2A loss impaired CD8A expression in all these seven cancer types (**Figures 4A, C, E, G, I, K, M**). The chemokine expression significantly decreased in the CDKN2A-deleted group as compared with the CDKN2A normal group (**Figures 4B, D, F, H, J, L, N**), indicating a similar regulatory mechanism in these cancers.

## Discussion

Melanoma is a malignant tumor with early metastasis, rapid progression, and high mortality rate. The role of immune cells, especially tumor-infiltrated T lymphocytes, has gained attention with the development of immunotherapy in recent years. T-cell infiltration in solid tumors is associated with a favorable outcome. However, infiltrated CD8+ T cells in cancer tissues are also characterized by impaired activity, increased apoptotic rate, and reduced cytokine production, termed as T-cell exhaustion. In the tumor microenvironment, cancer cells produce several immune-regulatory factors, such as adenosine, indoleamine 2,3-dioxygenase (IDO), and vascular endothelial growth factor A (VEGF-A) (47), which contribute to CD8+ T-cell exhaustion. Besides, the engagement of oncogenic pathways, such as Wnt/β-catenin activation, gain of MYC function, and NF-κB signaling, could reduce T-cell activation and infiltration (8). However, only few oncogenic pathways and their underlying mechanism in regulating T-cell infiltration have been studied. Our study provided several lines of evidence that CDKN2A loss was a potential indicator for identifying a T-cell-excluded subgroup in many cancer types.

First, CDKN2A loss highly correlated with T-cell marker expression and T-cell signature score in three melanoma cohorts and the clinical samples. The chemokine expression pathway was the most enriched pathway in the dys-regulated pathways between melanoma patients with normal CDKN2A versus deleted CDKN2A and melanoma patients with high CD8 versus low CD8 expression. Indeed, CCL3, CCL4, CCL5, CXCL9, CXCL10, and CXCL11 are all the recognized chemokines responsible for T-cell infiltration (48). Mechanically, the functional activation of CDKN2A results in a G1/S arrest which results in enhanced activation of p38 and NF-κB. Our founding was consistent with founding that G1/S arrest could increase chemokine expression (49).

Upon malignant transformation, the gain of genetic alternation resulted in a heterogeneity of cancer and oncogenic pathway activation that may cause dysregulation of immune cell and resulting in a T-cell exhausted microenvironment. The study found that a genetic event, CDKN2A deletion, directly correlated with CD8+ T-cell infiltration. Indeed, loss of CDKN2A was associated with melanoma invasion and progression, and cause immunotherapy resistance (35, 50). CDKN2A deletion repressed chemokine expression, and T-cell infiltration might be responsible for immunotherapy failure due to the loss of immunogenicity and resident immune cells. These findings were highly consistent with the concept that oncogenic pathways alternation modulates the immune avoidance and turn-on the “cold” tumor microenvironment (8, 51). Taken together, the findings suggested that the genetic deletion of CDKN2A led to low immune cell infiltration by reducing chemokine expression. Besides, not only melanoma but also urothelial BLCA, PAAD, LUAD, HNSCC, STAD, LUSC, and ACC showed reduced CD8A expression and CD8+ T-cell score in the CDKN2A-deleted group. However, it could not be concluded that the presence of CDKN2A directly induced T-cell infiltration due to the lack of *in vivo* experiments in this study. Further direct validation experiments are still needed in this regard.

In conclusion, the present study discovered a new oncogenic deletion of CDKN2A that regulated the chemokine expression and CD8+ T-cell infiltration in melanoma as well as other tumors, indicating that the genetic status of CDKN2A could be considered as a novel indicator of immune cell infiltration and exhaustion in cancer.

## Data Availability Statement

Publicly available datasets were analyzed in this study. These data can be found here: https://portal.gdc.cancer.gov and https://portals.broadinstitute.org/ccle.

## Ethics Statement

The studies involving human participants were reviewed and approved by the Pathology Department, Institute of Dermatology, Chinese Academy of Medical Sciences and Peking Union Medical College. The patients/participants provided their written informed consent to participate in this study.

## Author Contributions

ZZ did the bioinformatics analysis and conducted most of the experiments. HS did the clinical analysis of CD8 and CDKN2A expression in melanoma patients and revised the manuscript. JX confirmed the results. JX and ZZ wrote and revised the manuscript. All authors contributed to the article and approved the submitted version.

## Funding

This study was supported by grants from the Nanjing University (Grant Number: 22722001) and Fundamental Research Funds for the Central Universities (Grant Number: 3332019103).

## Conflict of Interest

The authors declare that the research was conducted in the absence of any commercial or financial relationships that could be construed as a potential conflict of interest.

## Abbreviations

ACC, adrenocortical carcinoma; AJCC, American Joint Committee on Cancer; ARF, alternative reading frames; BLCA, Bladder carcinoma; CCL, C-C Motif Chemokine Ligand; CCLE, Cancer Cell Line Encyclopedia; CXCL, C-X-C Motif Chemokine Ligand; DEGs, differentially expressed genes; DEL, Deleted; DMEM, Dulbecco’s modification of Eagle’s medium; FDR, False Discovery Rate; FPKM, Fragments Per Kilobase per Million; GSEA, gene set enrichment analysis; HNSCC, Head and neck squamous cell carcinoma; IDO, indoleamine 2,3-dioxygenase; IPA, ingenuity pathway analysis; IHC, Immunohistochemistry; KEGG, Kyoto Encyclopedia of Genes and Genomes; LSCC, Lung squamous cell carcinoma; LUAD, Lung adenocarcinoma; NOR, Normal; PAAD, Pancreatic adenocarcinoma; PI, Propidium Iodide; PVDF, polyvinylidene-difluoride; qRT-PCR, Quantitative Real-time PCR; RB, retinoblastoma; RSEM, RNA-Seq by Expectation-Maximization; SDS-PAGE, sulfate, sodium salt -Polyacrylamide gel electrophoresis; SKCM, skin cutaneous melanoma; STAD, Stomach adenocarcinoma; TCGA, The Cancer Genome Atlas; TPM, transcripts per million; VEGF-A, vascular endothelial growth factor A.
