# Supplementary material for: CDKN2A Deletion in Melanoma Excludes T Cell Infiltration by Repressing Chemokine Expression in a Cell Cycle-Dependent Manner
Source: Front Oncol. 2021 Mar 25;11:641077. doi: 10.3389/fonc.2021.641077 (PMC8027313; doi:10.3389/fonc.2021.641077)
Supplement: Supplementary file 1 [file DataSheet_1.docx]

Supplementary Material

# Supplementary Figures and Tables

## Supplementary Figures

**
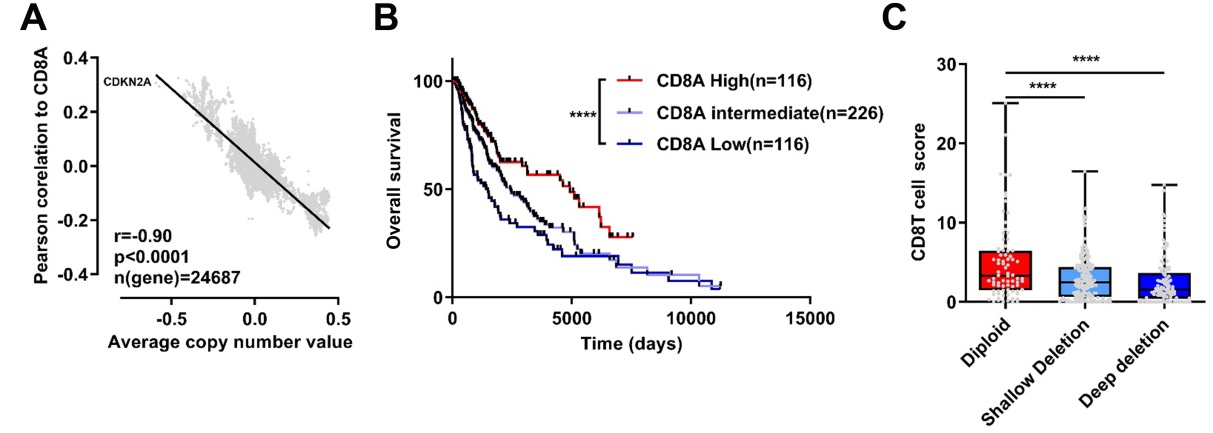
**

Supplementary Figure S1. Gene copy number alteration was associated with CD8A expression in melanoma. A. The association of gene copy number value and Pearson correlation of gene copy number to CD8A. B. Kaplan-Meier plot of overall survival of SKCM patients in TCGA-SKCM cohort with CD8A high, intermediated and low expression. C. CD8 T cell absolute score in CDKN2A diploid, shallow deletion and deep deletion.


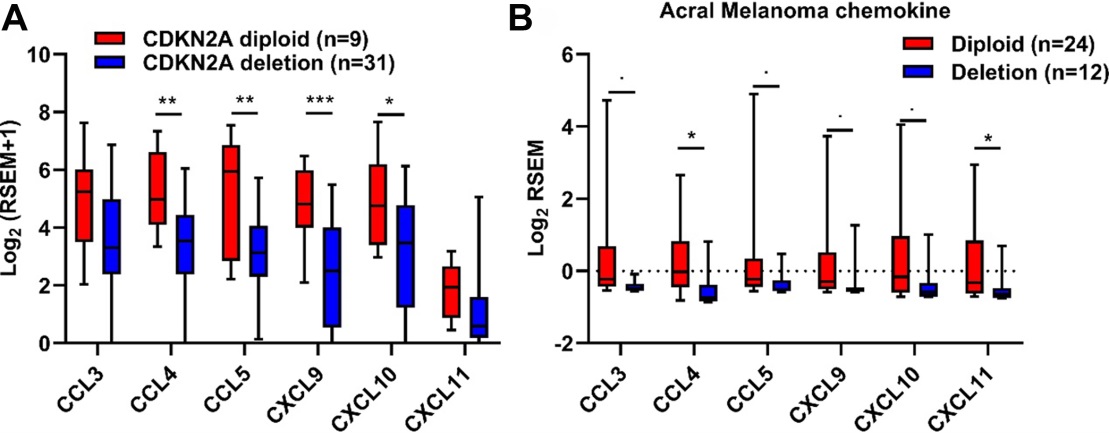
Supplementary Figure S2. Chemokine expression pathway alternation in CDKN2A diploid and deletion subgroups. A. Chemokine expression with CDKN2A diploid and deletion in DFCI cohort. B. Chemokine expression with CDKN2A diploid and deletion in TGEN cohort.


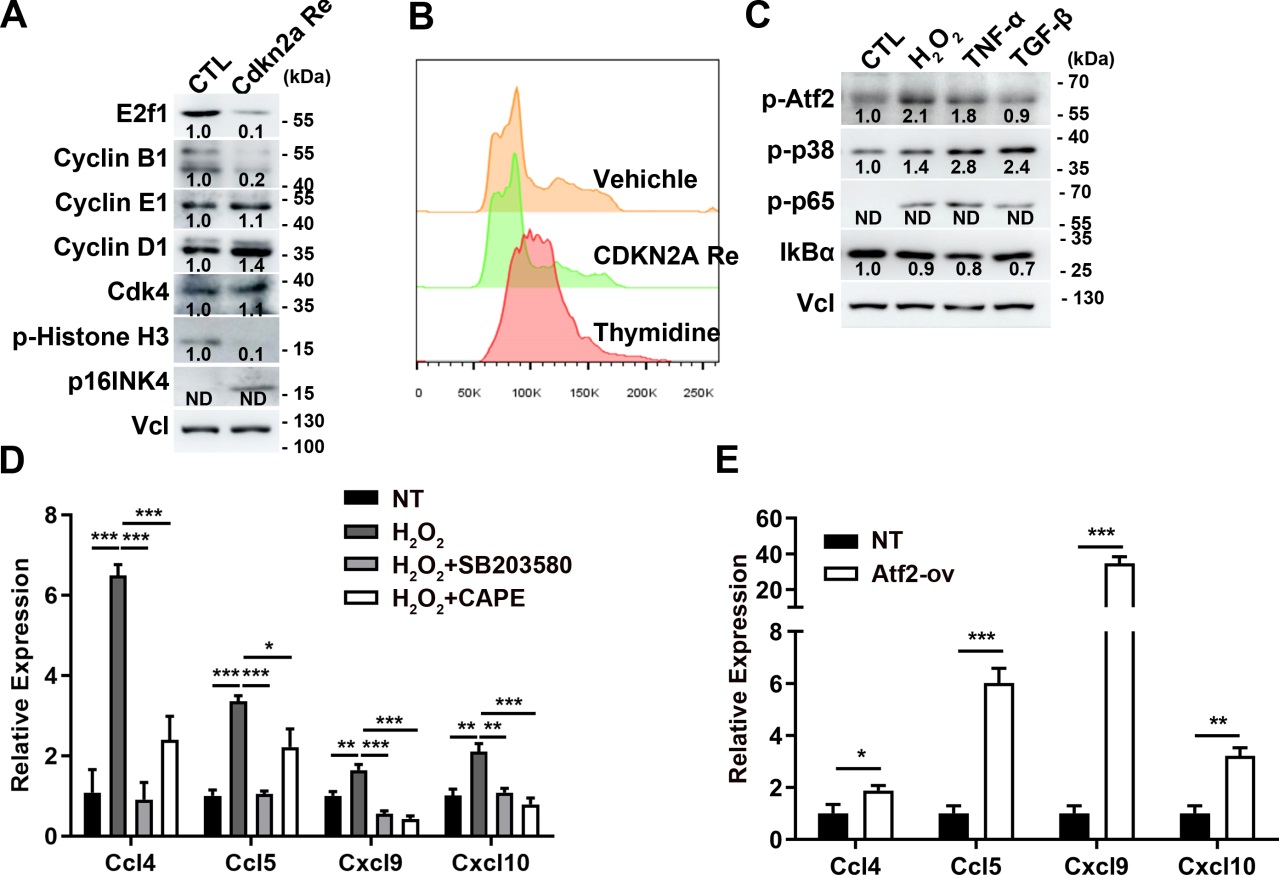


Supplementary Figure S3. H_2_O_2_ induce chemokine expression through activation of p38/MAPK and NF-κB signaling pathway. A. Cell cycle protein expression in control and Cdkn2a-re-expressed B16 cells. B. Represented cell cycle analysis by flow cytometry. C. The p38/MAPK pathway and NF-κB pathway protein expression in H_2_O_2_, TNF-α and TGF-β treatment B16 cells and control B16 cells. D. The chemokine Ccl4, Ccl5, Cxcl9 and Cxcl10 mRNA expression in B16 cells treated with H_2_O_2_, SB203580 (5 µM, the inhibitor of p38/MAPK pathway) or CAPE (10 µM, the inhibitor of NF-κB pathway). E. The chemokine Ccl4, Ccl5, Cxcl9 and Cxcl10 mRNA expression in control and Atf-2-overexpressed B16 cells.

## Supplementary Tables

Supplementary Table 1. Primer sequence.

| **Gene Name** | **Direction** | **Nucleotide sequences (5’ - 3’)** |
| --- | --- | --- |
| ***β-Actin*** | F | GGCCCAGAGCAAGAGAGGTATCC |
| ***β-Actin*** | R | ACGCACGATTTCCCTCTCAGC |
| ***Ccl3*** | F | TTCTCTGTACCATGACACTCTGC |
| ***Ccl3*** | R | CGTGGAATCTTCCGGCTGTAG |
| ***Ccl4*** | F | ATGAAGCTCTGCGTGTCTGCCC |
| ***Ccl4*** | R | AGCTGGCTTGGAGCAAAGACTGC |
| ***Ccl5*** | F | ATCTCTGCAGCTGCCCTCACCAT |
| ***Ccl5*** | R | GGCGGTTCCTTCGAGTGACAAAC |
| ***Cxcl9*** | F | GGAGTTCGAGGAACCCTAGTG |
| ***Cxcl9*** | R | GGGATTTGTAGTGGATCGTGC |
| ***Cxcl10*** | F | ATGAACCCAAGTGCTGCCGTCA |
| ***Cxcl10*** | R | CGCAGGGATGATTTCAAGCTTCC |
| ***Cxcl11*** | F | GGCTTCCTTATGTTCAAACAGGG |
| ***Cxcl11*** | R | GCCGTTACTCGGGTAAATTACA |
|  |  |  |

Supplementary Table 2. The association between CDKN2A, CD8A expression and clinicopathological characteristics in 20 melanoma patients.

| **Characteristics** | **n** | **CDKN2A** | | **p-value** | **CD8A** | | **p-value** |
| --- | --- | --- | --- | --- | --- | --- | --- |
|  |  | **Low(0~2)** | **High**  **(3~12)** |  | **Low**  **(0)** | **High**  **(1~2)** |  |
| **Melanoma type** |  |  |  |  |  |  |  |
| **In situ** | 5 | 1 | 4 | 0.735 | 1 | 4 | 0.800 |
| **Invasive** | 15 | 8 | 7 |  | 5 | 10 |  |
| **Gender** |  |  |  |  |  |  |  |
| **Male** | 8 | 4 | 4 | 0.734 | 2 | 6 | 0.571 |
| **Female** | 12 | 5 | 7 |  | 4 | 8 |  |
| **Age** |  |  |  |  |  |  |  |
| **≥60** | 10 | 6 | 4 | 0.603 | 4 | 6 | 0.112 |
| **<60** | 10 | 3 | 7 |  | 2 | 8 |  |
| **Ulceration** |  |  |  |  |  |  |  |
| **Yes** | 6 | 3 | 3 | 0.179 | 1 | 6 | 0.494 |
| **No** | 14 | 6 | 8 |  | 5 | 8 |  |
| **Breslow depth** |  |  |  |  |  |  |  |
| **≥2** | 11 | 5 | 6 | 0.949 | 4 | 7 | 0.851 |
| **<2** | 4 | 3 | 1 |  | 1 | 3 |  |
| **Clark’s level** |  |  |  |  |  |  |  |
| **I~II** | 6 | 2 | 4 | 0.904 | 2 | 4 | 0.779 |
| **III~V** | 14 | 7 | 7 |  | 4 | 10 |  |
